# Availability and use of Standards in vaccine development

**DOI:** 10.1038/s41541-023-00692-0

**Published:** 2023-06-30

**Authors:** Michael Selorm Avumegah, Giada Mattiuzzo, Anna Särnefält, Mark Page, Karen Makar, Janet Lathey, June Kim, Solomon Abebe Yimer, Danielle Craig, Ivana Knezevic, Valentina Bernasconi, Paul A. Kristiansen, Ingrid Kromann

**Affiliations:** 1 Coalition for Epidemic Preparedness Innovations (CEPI), Oslo, Norway; 2 Medicines and Healthcare products Regulatory Agency (MHRA), Potters Bar, UK; 3 Bill & Melinda Gates Foundation (BMGF), Seattle, USA; 4 National Institute of Allergy and Infectious Diseases (NIAID), Bethesda, USA; 5 World Health Organization (WHO), Geneva, Switzerland

**Keywords:** Vaccines, Drug development

## Abstract

Reference materials are critical in assay development for calibrating and assessing their suitability. The devasting nature of the COVID-19 pandemic and subsequent proliferation of vaccine platforms and technologies has meant that there is even a greater need for standards for immunoassay development, which are critical to assess and compare vaccines’ responses. Equally important are the standards needed to control the vaccine manufacturing processes. Standardized vaccine characterization assays throughout process development are essential for a successful Chemistry, Manufacturing and Controls (CMC) strategy. In this perspective paper, we advocate for reference material incorporation into assays and their calibration to International Standards from preclinical vaccine development through control testing and provide insight into why this is necessary. We also provide information on the availability of WHO international antibody standards for CEPI-priority pathogens.

## Background and current challenge

The need for harmonization and standardization in medical research is a concept which many scientists and researchers are aware. The International Organisation for Standardisation (ISO) was established in 1946 to introduce uniformity in safety and acceptance of services and products globally. ISO mission is broad and includes standards for global trade, economic growth indicators, health and safety, environment, and more^1^. The subsequent establishment of the International Council for Harmonization (ICH) in 1990 focused on ensuring medical or pharmaceutical products/devices for human use are safe, effective, and high-quality^2^. To achieve standardization, guidelines, and procedures were established for the validation and qualification of different processes and medical products. The World Health Organisation (WHO) has promoted the standardization of biological products, through the work of its Expert Committee on Biological Standardisation (ECBS) since 1947, generating written standards in the form of WHO Recommendations and Guidelines for many biological products including vaccines. This Committee also provides recommendations on the establishment of physical measurement standards referred to as WHO international reference standards^3^. WHO written and measurement standards for vaccines and other biologicals are provided as a basis for setting national regulatory requirements as well as a basis for WHO prequalification which is an important tool for global access to these products.

In response to the COVID-19 pandemic there has been an unprecedented proliferation of vaccine products and vaccine platforms. These approaches in antigen presentation ranged from more conventional inactivated / dead virus and recombinant protein to viral vectors, nucleic acids, nanoparticles and more.

The WHO released Target Product Profile for COVID-19 vaccines early on in the pandemic and follow up revision^4^. However, standards for innovative vaccine modalities, such as mRNA, have not been available for access until recently^5^. Vaccines are an important preventive measure for many diseases, and all aspects of its development from discovery, preclinical, clinical to commercial large-scale manufacturing and release to the market must have well-defined parameters for assessing safety, efficacy, and quality. The vaccine manufacturing process defines the product, and it is essential standardized assays are developed to characterize the product and define the process.

To assess and compare immune responses elicited by a vaccine throughout the development phases, assays and reference materials are essential. However, these are rarely available for novel pathogens and innovative vaccine platforms, as shown during the COVID-19 pandemic. Early establishment of reagents and standards for these assays is an important factor in evaluating the vaccine performance at various stages^6^.

In early-stage vaccine development, immune responses are assessed using laboratory analyses quantifying and characterizing antibodies and other immune markers found in serum or other body fluids following vaccination and comparing them to antibodies found in the serum of non-vaccinated individuals or those who have recovered from the target disease. Vaccine developers typically use a variety of different immunoassays with their own measuring units to report results, and they often use different biological materials as comparators. This is not a problem when assessing one vaccine, but it makes it challenging to compare immune responses between vaccines when assays are performed in different laboratories. Evaluation of vaccine-induced immune responses from clinical trials is critical to understand if a correlate or surrogate of protection can be identified^7^ and a protective threshold can be defined. In this perspective paper, we seek to inform the scientific community of the importance and role of reference materials in vaccine immune response evaluation and provide some insights for their use in manufacture and drug substance release.

## Reference material and WHO International Standards

In simple terms, reference material is established through robust processes and demonstrated to be fit for its intended use^8,9^. Therefore, such material, whether in purified form or complex matrix, is appropriate for assay calibration or quality control (QC) of an analytical procedure^10^. Table 1 provides insight for consideration on how and when reference materials should be used^11^. There is a hierarchy of reference material, based on uncertainty of measurement and intended use. The International Standard (IS) is the highest order of standard and the primary calibrant, with an arbitrary assigned unitage.

**Table 1:** Types of reference material.

Types of reference material	Usage
Assays	For the assessment of active pharmaceutical ingredients (APIs)
Degradation products	Used to establish a degradation curve for an analytical process
Process impurities	For the quantitation of process-related materials
Resolution	Used to determine assay performance or impurity method
Metabolites	For qualitative or quantitative determination of a metabolic process or product.

The development and implementation of IS for biological materials is a core function of the WHO that has an important impact on the high quality and consistent dosing of medicines used worldwide. These standards are widely used in the development, evaluation, standardization, and control of products in industry, by regulatory authorities, as well as in biological research in other scientific organizations.

WHO ISs are established by its ECBS with an assigned International Unit (IU). IS needs to be evaluated by those who use and are impacted by them^12,13^. Therefore, the establishment of any IS requires collaborative efforts of experts around the world. The process for the development of an IS is the critical factor that makes this the highest order of reference material^14^ and acts to ensure continuity of the IU usage through time and new technologies. Consequently, it is important to conserve the stocks of an IS and to this end, national authorities are encouraged to establish secondary standards. Similarly, manufacturers or research centers conducting numerous assays as part of their product development program usually establish their own in-house standards for routine use. The biological activities of such secondary preparations should be calibrated in IU by direct comparison with the respective IS.

## The role of CEPI in the development of International Standards for antibody

The Coalition for Epidemic Preparedness Innovations (CEPI) mission is to accelerate the development of vaccines and other biologic countermeasures against epidemic and pandemic threats so they can be accessible to all people in need. CEPI believes that human safety and quality must not be sacrificed on the crossroad of rapid development of vaccines and biologics. To achieve this, CEPI has launched several strategies and programs including supporting the production of IS for antibody in partnership with the Medicines and Healthcare products Regulatory Agency (MHRA), formerly known as the National Institute for Biological Standards and Control (NIBSC), a WHO collaborative center for biological standardization, and assay development for vaccine portfolios. CEPI is also open to more collaborations that align with the IS mission. Pathogens listed as a priority by WHO R&D blueprint and CEPI of which IS are available or are in preparation for research and vaccine development include Ebola^15^, Zika^16^, MERS-CoV^17^, Lassa^18^, Chikungunya (1502/19), SARS-CoV-2^19^, SARS-CoV-2 variants of concern^19^, Rift Valley fever^20^, Nipah, Mpox^21^, Marburg, Sudan Ebola, and SARS-CoV-1 (Table 2).

**Table 2:** International Standards for WHO/CEPI priority pathogens.

Name of pathogen	Description	NIBSC catalog number
Ebola virus	First International Standard for Ebola virus (EBOV) antibodies	15/262
Zika virus	First International Standard for anti-Asian-lineage Zika virus antibody	16/352
MERS-CoV	First WHO IS for Anti-MERS-CoV Immunoglobulin G (Human)	19/178
Lassa fever virus	First WHO IS for anti-Lassa virus antibodies	20/202
Chikungunya virus	First WHO International Standard for anti-chikungunya virus immunoglobulin G	1502/19, Paul Ehrlich Institute (PEI)
SARS-CoV-2	Second WHO International Standard for anti-SARS-CoV-2 immunoglobulin	21/340
SARS-CoV-2 variants of concern	First WHO International Standard for antibodies to SARS-CoV-2 variants of concern	21/338
Rift Valley fever virus	First WHO International Standard for neutralization assays and First WHO International Standard for binding assays	22/104_NT and 22/104_BD
Nipah virus	In progress	Expected to be available October 2023
Mpox	Working Reagent for anti-monkeypox virus antibodies	22/218
Marburg virus	In progress	Expected to be available 2024
Sudan virus	In progress	Expected to be available 2024
SARS-CoV (1)	In progress	

## Use of WHO IS during the COVID-19 pandemic

Within 6 weeks of COVID-19 being recognized as a pandemic, MHRA made available the research reagent NIBSC 20/130 prepared from convalescent plasma collected from one recovered patient with a high titer of anti-SARS-CoV-2 antibody^14^. This reagent offered a tool to assist assay development as a positive control.

In December 2020, the First WHO IS for anti-SARS-CoV-2 immunoglobulin (NIBSC 20/136)^22^ was established to facilitate the development and harmonization of serological assays to a common unitage^23^. These assays provide information on potential immune correlates of protection and are essential in supporting the clinical development of vaccines and therapeutics, as well as the seroepidemiological studies required to assess the impact of COVID-19. The assays broadly fall into two categories—virus neutralization assays and antibody binding assays such as enzyme-linked immunosorbent assays (ELISAs). Furthermore, 20/130 has been retrospectively calibrated to the IS and could be considered a secondary reagent; this approach allowed over 250 end users who received 20/130 to immediately convert their data into international standard unitage.

As part of technical assistance to users of WHO IS, a WHO manual for the preparation of International Reference Material for use as secondary standards in antibody testing has been developed and established by the ECBS in April 2022^24^. During the development of the Manual, several workshops were organized by WHO and CEPI to educate on the use of the International Standard.

Despite the relatively early availability of a research standard and subsequently a formally established IS, few researchers and vaccine developers have used these resources to report immunogenicity. In fact, none of the most advanced vaccine candidates used available reference material when reporting early clinical data or at the time of approval for emergency use. Efforts were put in place to encourage developers to make good use of the IS^14,25,26^. Uptake of IS 20/136, has been better by ELISA kit manufacturers than neutralization assay users. A possible explanation was that ELISA kit usually have an internal standard, which can be calibrated to the IS, while in neutralization assays results are mainly reported as absolute titers rather than relative to an internal control.

The multiple platforms explored for the COVID-19 vaccine highlighted the role for standards specific for a vaccine technology rather than a pathogen/disease, e.g., mRNA, vectored, etc. Such reagents would have expedited the transfer of assays undertaken by developers to manufacturers and the release of their COVID-19 vaccine at a large scale for global use. The use of common reagents would have facilitated the comparability studies between the various manufacturing sites, as well as aligning the release of different products at National control laboratories. The European Directorate for the Quality of Medicines & HealthCare (EDQM) and other authorities have published guidelines and monographs for official batch release.

Further collaboration on standardization of assays for Critical Quality Attributes (CQA), which are essential to deliver safe and efficacious vaccines^15^, could have contributed to faster delivery of more vaccine doses to the world.

## Why did it take so long, and what are the obstacles? View from industry

The challenge in the production of well-characterized reference materials is that the time taken to produce them does not correspond to immediate needs. Without a common standard available, vaccine developers will setup assays without the use of reference material, and it will require further time and resources to implement and validate the use of standards in their assays during clinical testing. This scenario often leads to a lack of uptake of the IS as seen in previous outbreaks like Ebola in 2013–2016 and Zika in 2015–2016. Solutions to this problem are the provision of less characterized standards, which might be back-calibrated to the IS, when one is available, and the retrospective calculation of the samples’ potency in IU. It is clear that the appropriate use of the WHO IS responds to the need for a common language when comparing immunogenicity results of clinical evaluation of candidate vaccines.

For COVID-19 the large number of kits detecting binding antibodies which have been developed represented a further obstacle to the harmonization as they were targeting slightly different viral antigens (RBD *vs* Spike protein as well as monomeric Spike *vs* trimeric). The emergence of various assays measuring cell-mediated immune responses further added complexities in the assessment of COVID-19 vaccine efficacy.

Thinking about preparedness for future pandemics, having reference material developed for diseases in the WHO R&D Blueprint and having infrastructure in place for collection of convalescent plasma or serum in every region of the world will save time. The development of monoclonal antibody cocktails can be a good alternative if convalescent serum is not available, as shown for Lassa disease^18,27^.

## How would the use of IS help? – View from regulators

Regulators typically review marketing application data in the context of a single developer application, where the key question is whether the presented data supports the product indication claim being sought. Nonetheless, this does not preclude the significance of adopting the IS. Preclinical and clinical assay results are among the key supporting data and can often be primary data when immunogenicity results are used as a surrogate marker of efficacy. This leads to a foundational question of whether the assay is suitable for its intended purpose. Once suitability is established, regulators can be assured that they may rely on the data for regulatory decision making. Early adoption of IS in bioanalytical method development and validation ensures the suitability of those assays through traceability to authentic sources of well-characterized materials of appropriate quality^2^. Further, in cases where surrogate markers of efficacy are used as the primary approval data, the use of IS simplifies post-authorization actions for monitoring and pharmacovigilance in the critical window while effectiveness is confirmed.

Of secondary importance, regulatory authorities often present data to technical advisory groups, who have the responsibility to make recommendations for national use guidelines, evaluation of benefit-risk on an individual or population level, and government purchasing actions. These technical advisory groups have the authority to make recommendations based on a direct comparison of all the available products. Reporting assay results in IU simplifies aspects of these comparisons, as the data under consideration can be considered like-for-like.

## The role of an IS in determining correlates of protection

Evaluation of the immunogenicity of a vaccine may lead to the identification of a correlate of protection (CoP). However, identification of a CoP that can be applied across vaccines requires the comparison of immunological data from different clinical trials and is often confounded by differences in assays and numerical readouts. For one specific disease, should neutralizing antibodies be found to provide a surrogate of protective response, the expression of neutralizing antibody responses in IU/mL is essential to gather a consensus from across vaccines of several clinical trials and other studies on the titer required for protection^22–25^.

## Other initiatives to harmonize results

While the use of the WHO IS increases the comparability of the results from different studies, it is not sufficient to standardize assays and reliably obtain the same results from different laboratories. In response to the COVID-19 pandemic, the CEPI Centralized Laboratory Network was launched for the harmonization of immune response assessment across COVID-19 vaccine candidates. CEPI centralized laboratories achieve harmonization of the results from different vaccine clinical trials with the use of common standard operating procedures and the same critical reagents, including a working standard calibrated to the IS. CEPI Centralised Laboratory Network will expand its scope by tackling immunological testing of vaccine against other diseases going forward from 2023^26^.

CEPI has developed a CMC framework to support vaccine developers throughout the product development phases, advising to establish methods for characterization and stability measurement in the very early stages in order to be able to compare the products used for the early toxicology and safety studies with the products used in the pivot studies.

## Call for action

All vaccine developers should be asked to report antibody results from clinical trials relative to the WHO IS when such material has been established and is available. Due to time constraints, this might not always be feasible in early pre-clinical or clinical development, but bridging of results to IU later is of critical importance for the interpretation of the results in the context of the evaluation of immune response to vaccines in the common language of IU.

It would be of benefit if the use of International Standards would be highlighted by scientific journals, peer reviewers and regulatory authorities. Access to IS is open, but getting access to secondary standards, which can be used on each test plate and back-calibrated to the International Standard is also needed. With regards to vaccine manufacture, to standardize control testing, industry engagement is needed to establish and use reference material.

## Conclusion

Our main aim has been to provide information on the availability of several international antibody standards for WHO/CEPI priority pathogens to support vaccine development efforts and highlight the importance of preparing standards and standardized methods for measurement of the vaccine effect as a tool both in process and clinical development. We also advocate for deliberate consideration and establishment of reference standards for characterization of the products for quality assessment and control testing.
